# German Public Support for Tobacco Control Policy Measures: Results from the German Study on Tobacco Use (DEBRA), a Representative National Survey

**DOI:** 10.3390/ijerph15040696

**Published:** 2018-04-07

**Authors:** Melanie Boeckmann, Daniel Kotz, Lion Shahab, Jamie Brown, Sabrina Kastaun

**Affiliations:** 1 Institute of General Practice, Addiction Research and Clinical Epidemiology Unit, Medical Faculty of the Heinrich-Heine-University, 40227 Düsseldorf, Germany; daniel.kotz@med.uni-duesseldorf.de (D.K.); sabrina.kastaun@med.uni-duesseldorf.de (S.K.); 2 Department of Behavioural Science and Health, University College London, London WC1E 6BT, UK; lion.shahab@ucl.ac.uk (L.S.); jamiebrown10@gmail.com (J.B.)

**Keywords:** tobacco control, policy, epidemiology, public opinion, survey

## Abstract

Smoking prevalence in Germany remains high at approximately 28%. We assessed public support for tobacco legislation and associations between level of support and smoking and socio-demographic characteristics. Data from 2087 people were collected as part of the German Study on Tobacco Use (“DEBRA”): a nationally representative, face-to-face household survey. Public support was measured on total ban of sale, raising the minimum age for sales, taxation of tobacco industry sales, research into e-cigarettes, and ban of smoking in cars when children are present. Associations were assessed with multivariate logistic regression. Over 50% of the German population support taxing industry profits (57.3%) and assessing e-cigarettes as an aid to quit smoking (55.5%). Over 40% support raising the legal age of sale (43.1%), and 22.9% support a total ban on tobacco sales. A smoking ban in cars when children are present was most popular (71.5%), even among current smokers (67.0%). There is public support for stricter tobacco control measures in Germany. A smoking ban in cars when children are present could be a feasible policy to implement.

## 1. Introduction

Tobacco smoking is highly addictive and a major risk factor for mortality and morbidity. Compared with other high-income European countries, smoking prevalence in Germany is high at approximately 28% [1], and 14% of the country’s mortality can be attributed to smoking [2]. More than 120,000 people in Germany die from tobacco-related illness each year [2], and second-hand smoke exposure for children and adolescents, while reduced over the past decades, remains a problem [3], particularly in families with lower incomes and a poorer educational background [4]. It is estimated that up to 27% of all hospitalisations for diagnoses of asthma, respiratory diseases, or otitis media in children younger than five years can be attributed to passive smoking [5]. Smoking remains a significant public health issue in Germany; yet tobacco control in Germany continues to be contested. Germany was among the ten countries with the largest population of smokers in 2015, with an age-standardised estimated number of 7.1 million female and 9.2 million male smokers [6]. The tobacco industry continues to be highly active in German tobacco-related legislation [7,8,9]. Owing to Germany’s strong economic position within the E.U., the country’s tobacco control efforts are also interesting to other European countries.

Tobacco control legislation is a cornerstone of the World Health Organization (WHO) Framework Convention on Tobacco Control (FCTC), and Germany, together with 179 other countries, has ratified the convention, thus pledging to implement laws and measures to decrease tobacco use prevalence [10]. Germany has a minimum age of sale for tobacco products of 18 years, tobacco products are taxed, and pictorial health warnings were introduced in 2016 [11], but comprehensive implementation has not yet been achieved [12]. Advertisement of tobacco products outdoors, at points of sale, and in cinemas and indoor smoking in certain bars and pubs are all still allowed [9]. Other European countries have made substantial progress in protecting non-smokers from second-hand smoke exposure. A number of European nations, including Italy [13], Finland [14], England [15], Wales [16], and Ireland [17] have introduced laws to ban smoking in private vehicles when minors are present. Even though the German Medical Association and political representatives, such as the Drug Commissioner of the Federal Government of Germany, have expressed an urgent need for such a ban in Germany [18], smoking in cars with children is still allowed.

Treatment for tobacco addiction is not reimbursed by German health insurance companies, forcing patients to bear the costs themselves [19,20]. This may influence the likelihood of smokers actually trying to stop smoking with evidence-based methods [21]. Indeed, most smokers in Germany still try to quit smoking unaided or with the use of non-evidence-based treatments [22,23] and therefore have a very low chance to succeed. Electronic cigarettes (EC) could be promising devices to aid in smoking cessation, partly because they are relatively cheap and less harmful [24,25,26] compared with regular cigarettes and easily accessible even for those on lower incomes [27]. However, e-cigarette use is not without risk [28,29,30], and information on the long-term effects of use is currently still lacking [31]. A recent review on the impact of e-cigarettes on smoking cessation showed that good-quality studies assessing the topic are often lacking, yet the few high-quality studies we have do seem to indicate potential for e-cigarettes as a cessation tool [32,33]. These findings echo those of previous Cochrane reviews [27,31]. There is widespread concern about e-cigarettes acting as a gateway to cigarettes for younger people. Numerous prospective studies have established that adolescents who have tried e-cigarettes are more likely to report subsequent smoking after adjustment for important factors [25,34,35]. There is disagreement on the extent to which the adjustment for the likely common liability is sufficient [36]. Reassuringly, there is not yet population-level evidence in countries where e-cigarettes have become popular of long-term declines in cigarette smoking uptake being undermined [26]. Further surveillance and investigation is required, including context-specific studies on the German healthcare system. Views of the German public on e-cigarette safety and efficacy as cessation aid have also not been studied conclusively.

Data from the Tobacco Control Ranking Scale in 2016 show that Germany is at place 34 out of 35 countries in successful tobacco control implementation in Europe [9]. As studies have shown that price increases and taxation, limited access to tobacco products, second-hand smoke exposure restrictions, and plain packaging all contribute to lowering smoking prevalence in European countries [9], less advanced tobacco control and high smoking prevalence are correlated [37]. Tobacco control is thus a promising avenue for better public health, which also applies in Germany.

Policymakers may be convinced that more restrictive legislation on tobacco use and access is unpopular among their constituency, which could be influencing their policies. Public support for public health measures is therefore an interesting item to measure for a better understanding of links between being personally affected and one’s endorsement of legislative changes. Findings from the English Smoking Toolkit Study, for example, show that broad public support for price raises of cigarettes or even a total ban on tobacco product sales exists, even among current smokers [38,39]. Similarly, a representative survey study from the U.S. found widespread support for large cigarette pack warning labels among both smokers and non-smokers [40]. Recent findings on public support for the 2013 smoking ban in restaurant and bars in the German federal state of North-Rhine Westphalia suggest that the state’s population largely agrees with the restriction and supports limiting second-hand smoke exposure [41]. Schaller et al. similarly found evidence of support among the German population for non-smoking legislation in public places, in particular regarding smoking in cars when children are present [42]. Considering Germany’s high level of smoking prevalence, limited political commitment to full FCTC implementation, and subsequent high burden of disease, more representative data on public perceptions of tobacco control are needed to improve our understanding of opportunities for structural prevention. This article reports representative survey data on the public support for five possible legislative changes in Germany: a total ban on the sale of tobacco products, raising the minimum age for tobacco sales, taxation of tobacco industry sales, research into e-cigarettes as tobacco cessation aids, and legislation to curb children’s second-hand smoke exposure in cars.

## 2. Materials and Methods

### 2.1. Data and Participants

Data were taken from the German Study on Tobacco Use [23] (DEBRA: “**DE**utsche **B**efragung zum **RA**uchverhalten”), an ongoing representative household survey on tobacco use in Germany. DEBRA has been registered in the German Clinical Trials Register (registration number DRKS00011322), and the study protocol has been published elsewhere [43]. Details about recruitment and sampling strategies for the DEBRA study are described in the study protocol [43]. In brief, every two months a new representative sample of ~2000 respondents aged 14 years and older completes a computer-assisted, face-to-face survey. A multi-stage, multi-stratified random probability sampling approach is used. Fieldwork is conducted by the market research institute Kantar Health Munich, Germany, and per wave up to 280 trained interviewers conduct on average 7 interviews each. Respondents report data on demographics and the use of tobacco and EC, with smokers additionally answering detailed questions about smoking behaviour, quit attempts, exposure to health professionals’ advice on quitting, and use of cessation aids [1]. Questions on support for tobacco control policies were added to wave two of the survey in September 2016. A total of 2087 respondents participated in this wave of the survey.

### 2.2. Measures

#### 2.2.1. Policy Questions

Respondents were asked to indicate whether they supported the following statements [43]:

1. “The sale of cigarettes and tobacco in Germany should be banned completely within the next 10 years.”
2. “The legal age of sale of cigarettes and tobacco in Germany should be raised from 18 to 21.”
3. “Tobacco industry sales should be taxed in order to use the money to address problems caused by tobacco (e.g., health issues, environmental problems, etc.).”
4. “It should be assessed whether e-cigarettes are safe and effective in assisting smokers to quit.”
5. “When minor children are in the car, smoking inside the car should be banned and subjected to punishment.”

Answering options were:

1. “Strongly support”
2. “Tend to support”
3. “No opinion either way”
4. “Tend to oppose”
5. “Strongly oppose”
6. “No answer”

We re-coded answering options 1 and 2 into “agree”, 4 and 5 into “disagree”, and 3 as “undecided” for the descriptive statistics. For the regression analyses, we dichotomised answers into “agree” (strongly support, tend to support) and “don’t agree” (no opinion, tend to oppose, strongly oppose). This allowed for comparison of our results with those of prior analyses of the English Smoking Toolkit Study [38,39].

#### 2.2.2. Socio-Demographic Characteristics

Self-reported data on age, sex, education, respondent and net household income, and employment status were collected. We measured socio-economic status with the two most relevant indicators in the regression model: net income, categorised as 1 = less than 1000 €/month 2 = 1000–less than 2000 €/month, 3 = 2000–less than 3000 €/month, 4 = 3000–less than 4000 €/month, 5 = 4000–less than 5000 €/month 6 = more than 5000 €/month, and education, categorised from lowest to highest attainment as 1 = no qualification, 2 = junior high school equivalent (“Hauptschulabschluss”), 3 = secondary school equivalent (“Realschulabschluss”), 4 = advanced technical college equivalent (“Fachhochschulreife”), 5 = high school equivalent (“Allgemeine Hochschulreife”).

#### 2.2.3. Smoking Characteristics

Participants were categorised as current smokers if they smoked cigarettes (including hand-rolled) or any other combustible tobacco (e.g., pipe or cigar) daily or occasionally; as ex-smokers if they had stopped in the last year or more than a year ago; and as never smokers if they had never been a smoker (i.e., never smoked for a year or more). Current smokers were asked to also indicate the number of cigarettes smoked daily, weekly, or monthly, whether they made a quit attempt in the past year, and about their motivation to stop smoking using the translated and culturally adapted German version of the Motivation to Stop Smoking Scale (in German: “Motivation zum Rauchstopp Skala“, MRS) [43,44].

### 2.3. Analyses

We carried out descriptive statistics to characterise the sample and to establish prevalence rates for support per policy question, reported for the total sample and separately for current smokers, ex-smokers, and non-smokers. The sample was weighted to be representative of the German population for calculation of the primary outcome prevalence rates (support for policy measures). Associations between smoking status, socio-demographic characteristics, and support for policy measures were assessed using multivariate logistic regression with the dichotomous dependent variable “agree on a policy statement” (yes/no). Several pre-defined potential confounders were considered in the analyses: sex, age, net household income, education, smoking status, and, for a subgroup analysis of current smokers, smoking characteristics, such as attempts to quit during the past year, number of cigarettes smoked per day, and their current motivation to stop smoking. These associations were examined with unweighted data. Our approach to all analyses was exploratory.

Respondents with missing data on their smoking status were excluded from all analyses (*n* = 25, 1.1% from the total sample). A total of 2062 respondents were thus included in the analysis. Furthermore, respondents who did not want to answer questions regarding the policy statements or those with missing data on their educational level or their attempts to quit smoking were excluded only from the multivariate logistic regression analyses (question 1 = 215 missings (10.4%), question 2 = 185 missings (9%), question 3 = 194 missings (9.4%), question 4 = 240 missings (11.6%), question 5 = 162 missings (7.4%)).

## 3. Results

Of the total sample of 2062 respondents, 586 (28.1%, 95% confidence interval (95% CI) = 26–30%; unweighted) were current smokers, 369 (17.7%, 95% CI = 16–19%) ex-smokers, and 1107 (53.0%, 95% CI = 51–55%) never smokers. Baseline characteristics, including socio-demographic characteristics, are presented in Table 1. The mean age was 51.8 years (standard deviation (SD) = ±20), and 1070 (51.9%) of the respondents were female. Smoking characteristics of the subsample of current smokers are presented as unweighted data in Table 2.

### 3.1. Public Support for Tobacco Policy Measures

**Figure 1:**
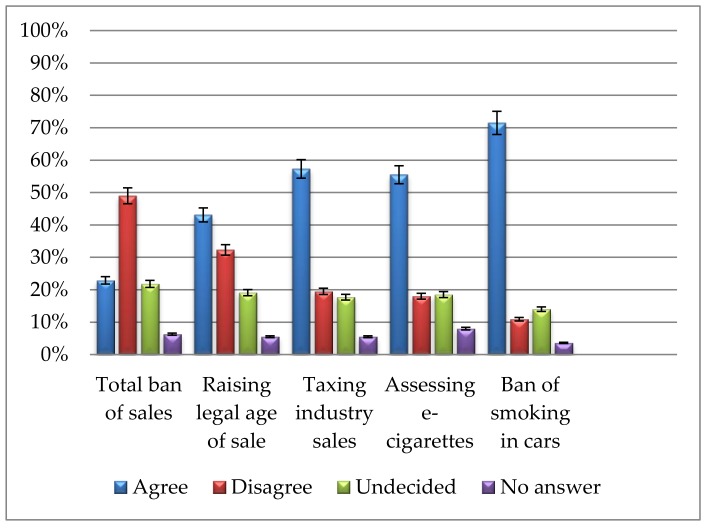
Proportion (and 95% confidence intervals) of public support for the five tobacco policy measures with the re-coded answer options agree (strongly support, tend to support), disagree (strongly oppose, tend to oppose), undecided (no opinion either way), or no answer (*n* = 2062 respondents).

Results, weighted to be representative for the German population, are presented in Figure 1. Of the total sample, 22.9% (95% CI = 21.1–24.8%) would support a total ban on the sale of tobacco and cigarettes within the next ten years, 21.8% (95% CI = 20.0–23.6%) were undecided on that matter, and almost half of the sample (49.0%, 95% CI = 46.8–51.2%) disagreed with the proposed statement.

Raising the legal age of sale from 18 to 21 would be supported by 43.1% (95% CI = 41.0–45.3%) of the respondents, whereas 32.3% of the respondents (95% CI = 30.3–34.4%) disagreed with the proposed statement on such a policy change.

A large majority of respondents (71.5%, 95% CI = 69.5–73.5%) would support a total ban in cars when minors are present; only 10.9% (95% CI = 9.5–12.3%) disagreed with this statement.

Among the subsample of current smokers (weighted subsample *n* = 542), 9.9% (95% CI = 7.4–12.5%) would support a total ban on the sale of tobacco and cigarettes within the next ten years. Also, 10.3% (95% CI = 7.8–12.9%) were undecided if they would support such a total ban, and nearly three-quarters of the sample (74.0%, 95% CI = 70.5–77.9%) disagreed with the proposed statement.

More than half of the smokers were either supportive (34.6%, 95% CI = 30.7–38.7%) or undecided on the question (18.4%, 95% CI = 15.2–21.7%) of whether the legal age of sale should be raised from 18 to 21.

Half of smokers (50.1%, 95% CI = 46.0–54.4%) agreed on the importance of assessing the effectiveness and safety of e-cigarettes as an aid to quit smoking tobacco, while 21.7% (95% CI = 18.3–25.2%) and 24.3% (95% CI = 20.7–28.0%) were undecided or disagreed with this statement, respectively. 

Comparable with the total sample, the majority of current smokers (67.0%, 95% CI = 63.0–70.9%) would support a total ban in cars when minors are present; only 14.4% (95% CI = 11.4–17.3%) disagreed with this statement.

### 3.2. Results from the Regression Analysis

Results of the unweighted multivariate logistic regression for each of the five policy statements are presented in Table 3 (for the total sample), Table 4 (for the subgroup of current smokers), and Table 5 (for the subgroup of ex- and never-smokers). We found no associations between sex, education, or household income and support for policy measures. With increasing age (in 10-year-units), overall support for these policy recommendations decreased.

#### 3.2.1. Support for a Total Ban on Tobacco Sales

Support for a total ban on tobacco sales was associated with smoking status. Never-smokers and ex-smokers were significantly more likely to indicate their support for a total ban on tobacco sales than current smokers (odds ratio (OR) 5.26, CI 3.80–7.28 and OR 3.48, CI 2.37–5.11, respectively).

Among current smokers, respondents who had not tried to quit in the past year were less likely to support a total ban on sales than respondents who indicated that they had tried to quit (OR 0.20, CI 0.10–0.40). Motivation to quit smoking did not show significant associations with support for a total ban.

#### 3.2.2. Support for Raising the Legal Age of Sale

Support for raising the legal age of sale from 18 to 21 was also dependent on smoking status: never-smokers and ex-smokers were more likely to support this measure than current smokers (OR 2.73, CI 2.17–3.46 and OR 1.75, CI 1.30–2.35, respectively).

Among current smokers, higher levels of motivation to stop smoking (OR 1.11, CI 1.02–1.20) were associated with greater support of this policy.

#### 3.2.3. Support for Special Taxation of Tobacco Industry Profits

Men were marginally less likely to support taxation of industry profits than women (OR 0.81, CI 0.66–0.99). Never- and ex-smokers were more likely to support the proposed measure than current smokers (OR 4.16, CI 3.29–5.24 and OR 3.48, CI 2.59–4.66, respectively).

Among current smokers, all income categories had a lower likelihood of supporting taxation compared with the highest income earning group (see Table 4).

#### 3.2.4. Support for Assessing the Effectiveness and Safety of EC as Cessation Aids

Never-smokers and ex-smokers had 50% and 40% higher odds, respectively, of supporting assessing the effectiveness and safety of EC compared with current smokers (OR 1.50, CI 1.19–1.87 and OR 1.39, CI 1.04–1.85, respectively). Within the subsample of current smokers, no associations were found.

#### 3.2.5. Support for Ban on Smoking in Cars When Children Are Present

Never-smokers were more likely than current smokers to support the ban on smoking in cars (OR 1.81, CI 1.41–2.31); however, no such association could be found when comparing ex-smokers with current smokers. Among the smoker subsample, men were about half as likely to support a ban on smoking in cars when minors are present as women (OR 0.55, CI 0.37–0.84).

## 4. Discussion

Our results indicate that a substantial share of the German population would support stricter tobacco control policies than those currently implemented. Of the five suggested policy measures, three were supported by more than half of the population: taxing industry sales, assessing e-cigarettes, and ban on smoking in cars when children are present. Over 40% also supported raising the legal age of sale. Twenty percent of the population would support a total ban on tobacco products in Germany within ten years. 

A total ban on the sale of tobacco is a quite intrusive measure yet appeals to 20% of the population. Among smokers, however, only 10% would support a total ban of sales. The proposed measure of banning smoking in cars when a child is present received the highest—70%—support in the German population. Even among the subsample of smokers, 67% indicated their support for a smoking ban in cars. A similarly high level of support for this policy has previously been reported for Germany [42], and also for the U.K., Australia, Canada, and the United States [45]. Tobacco control measures are generally also supported in the European population [46,47]. 

Smoking status was associated with whether or not a person agrees with a policy measure. Never-smokers and ex-smokers were consistently more likely to support a control policy than current smokers across all proposed measures. Motivation to quit only showed a significant association with support for one measure: raising the legal age of sale. Motivation to quit seems to play a less important role in shaping German smokers’ opinion towards more restrictive tobacco control policies.

Supporting a tobacco control measure was generally not significantly associated with sex, education, or income, with a few exceptions. With increasing age (in 10-year-units), overall support for these policy recommendations decreased. The lack of a clear socio-demographic pattern for supporting policies echoes findings from England on price increases of tobacco [39], and suggests that tobacco control objectives are shared across social gradients and gender. Participants who state that they are undecided on whether to support a policy may be susceptible to information campaigns or targeted health education. It would be worth exploring how to target these groups specifically.

Comparing our study with findings from the U.K. Smoking Toolkit Study, our results show that there is currently less support for a total ban in Germany (22%) than in England in 2008 (44%) [38]. Given the associations between support and smoking status, some of the difference is likely associated with the higher smoking prevalence in Germany. There is also higher public acceptance of smoking bans in public places in the U.K. [48]. Other studies from the European context have similarly shown that overall, public support for tobacco control exists [49], including for smoking bans in outdoor spaces surrounding hospitals or schools [50] as well as for total smoking bans [51]. Support for price increases varies: whereas the English public seems to agree with tax increases [39], support in European countries with lower GDPs has declined in past years [49]. Our results do not show associations with socio-demographic characteristics, yet a recent study by Filippidis et al. [52] raises the possibility of associations between political views and support for tobacco control. Further insights into reasons behind these variations by European country might be useful for coherent European tobacco control strategies.

As Germany is a federal country, a coordinated policy approach across state lines might be needed to fully implement additional tobacco control measures. For these analyses, we did not assess responses by individual states as the sample sizes per state were too small for statistical analyses. A total ban on sales within a country in Europe also needs to consider European regulations, and must aim to avoid unintended negative consequences of a complete ban. Even less restrictive tobacco control measures have been shown to contribute to reduced smoking prevalence [53,54], which in turn may lead to decreased public expenditure on tobacco-related morbidity, fewer tobacco-related deaths [55], and better population health. Policymakers in Germany could therefore consider first implementing those measures garnering wider support in the population.

This study has some important limitations. We were only able to analyse data from one wave of the survey that included the policy support questions. It would be interesting to compare results longitudinally to assess whether public support changes over time. We were interested in broad attitudes towards tobacco control policies that are not currently implemented in Germany. How the public would react to the actual implementation of measures cannot be inferred from our results. We also did not provide participants with suggestions on how such measures would be implemented: the hypothetical nature of these policies may have influenced responses. We were unable to include more than five policy-related questions into the survey wave so as to not overburden participants. Future survey waves would ideally also elicit information on support for additional measures, such as indoor smoking bans in public housing, which have recently been implemented in the United States [56].

Despite these limitations, our results suggest a first, feasible step forward in tobacco control in Germany would be instituting a smoking ban in cars when children are present. Starting with a seemingly non-controversial policy implementation may prepare the way for future implementation of more contested measures.

## 5. Conclusions

Public support for additional tobacco control measures is moderately high in Germany. A smoking ban in private vehicles if children are present is supported by a majority of the population (over 70%), even among smokers (67.0%). These findings indicate that implementation of such a policy that already exists in other European countries might also be feasible in Germany.

## Figures and Tables

**Table 1:** Unweighted baseline characteristics of the total sample, and by smoking status.

	Total Sample (*n* = 2062; 100%)	Current Smoker (*n* = 586; 28.4%)	Ex-Smoker (*n* = 369; 17.9%)	Never Smoker (*n* = 1107; 53.7%)
Age, years (mean ± SD)	51.8 ± 19.8	47.1 ± 17.2	58.4 ± 17.5	52.1 ± 21.1
Sex				
Female	1070 (51.9%)	271 (46.2%)	143 (38.8%)	656 (59.3%)
Male	992 (48.1%)	315 (53.8%)	226 (61.2%)	451 (40.7%)
Education ^†^				
High school equiv.	479 (23.2%)	110 (19.2%)	85 (23.2%)	284 (27.4%)
Adv. tech. college equiv.	133 (6.5%)	28 (4.9%)	30 (8.2%)	75 (7.2%)
Secondary school equiv.	686 (33.3%)	230 (40.1%)	116 (31.7%)	340 (32.8%)
Junior high school equiv.	646 (31.3%)	193 (33.6%)	130 (35.5%)	323 (31.1%)
No qualification	33 (1.6%)	13 (2.3%)	5 (1.4%)	15 (1.4.5%)
Household income				
>€5000/per month	134 (6.5%)	26 (4.4%)	27 (7.3%)	81 (7.3%)
€4000–5000/per month	128 (6.2%)	31 (5.3%)	24 (6.5%)	73 (6.6%)
€3000–4000/per month	369 (17.9%)	96 (16.4%)	67 (18.2%)	206 (18.6%)
€2000–3000/per month	557 (27.0%)	164 (28.0%)	106 (28.7%)	287 (25.9%)
€1000–2000/per month	638 (30.9%)	173 (29.5%)	117 (31.7%)	348 (31.4%)
<€1000/per month	236 (11.4%)	96 (16.4%)	28 (7.6%)	112 (10.1%)

Data are presented as number (%), unless otherwise stated. ^†^ German equivalents to education levels listed in table from highest to lowest: high school equivalent = “Allgemeine Hochschulreife”, advanced technical college equivalent = “Fachhochschulreife”, secondary school equivalent = “Realschulabschluss”, junior high school equivalent = “Hauptschulabschluss”.

**Table 2:** Unweighted smoking characteristics of current smokers.

	Current Smokers Only (*n* = 586)
Cigarettes smoked per day (mean ± SD)	15.3 ± 9.0
Made at least one quit attempt last year	140 (23.9%)
Motivation to stop smoking (MRS) [43]	
Do not want to stop smoking	268 (45.7%)
Should stop but do not really want to	139 (23.7%)
Want to stop but have not thought about when	52 (8.9%)
Want to stop but have not decided when	51 (8.7%)
Really want to stop and hope to soon	43 (7.3%)
Really want to stop and intend to in the next 3 months	7 (1.2%)
Really want to stop and intend to in the next month	6 (1.0%)

Data are presented as number (%), unless otherwise stated.

**Table 3:** Multivariate associations with support (agree/totally agree) for the policy statements in the total sample (*n* = 2062).

	Banning Sale of Tobacco	Raising Legal Age of Sale from 18 to 21	Taxing Tobacco Industry Sales, to Address Tobacco-Related Problems	Assessing Effectiveness and Safety of E-Cigarettes	Banning Smoking in Cars When Minors Are Present
Smoking status					
Current smoker (ref.)	1	1	1	1	1
Ex-smoker	3.48 (2.37–5.11) ***	1.75 (1.31–2.36) ***	3.48 (2.59–4.66) ***	1.39 (1.04–1.85) *	1.29 (0.95–1.74)
Never-smoker	5.26 (3.80–7.28) ***	2.73 (2.17–3.46) ***	4.16 (3.30–5.24) ***	1.50 (1.20) ***	1.81 (1.42–2.31) ***
Age, 10-year units ^a^	0.98 (0.92–1.04)	0.95 (0.89–1.00) *	1.02 (0.96–1.08) *	0.94 (0.89–0.99) *	0.94 (0.89–1.00) *
Sex					
Female (reference)	1	1	1	1	1
Male	0.83 (0.66–1.03)	0.84 (0.69–1.02)	0.81 (0.66–0.99) *	0.88 (0.72–1.07)	0.89 (0.72–1.10)
Education ^†^					
High school equiv. (ref.)	1	1	1	1	1
Adv. tech. college equiv.	1.59 (1.00–2.51)	1.19 (0.79–1.801)	0.86 (0.56–1.33)	1.15 (0.75–1.75)	1.28 (0.79–2.07)
Secondary school equiv.	1.15 (0.85–1.56)	1.14 (0.88–1.48)	0.81 (0.62–1.07)	0.79 (0.61–1.02)	0.97 (0.73–1.29)
Junior high school equiv.	1.31 (0.94–1.83)	1.31 (0.99–1.74)	0.65 (0.49–0.88) **	0.80 (0.60–1.06)	1.10 (0.81–1.51)
No qualification	0.89 (0.33–2.39)	2.00 (0.91–4.40)	0.99 (0.42–2.32)	0.37 (0.16–0.85) *	0.86 (0.37–2.00)
Household income					
€>5000/per month (ref.)	1	1	1	1	1
€4000–5000/per month	1.13 (0.62–2.05)	1.08 (0.64–1.82)	0.68 (0.39–1.19)	1.18 (0.71–1.97)	0.78 (0.43–1.42)
€3000–4000/per month	0.81 (0.49–1.35)	1.10 (0.71–1.69)	0.72 (0.45–1.16)	1.13 (0.74–1.73)	0.75 (0.45–1.23)
€2000–3000/per month	1.14 (0.71–1.83)	1.11 (0.73–1.68)	0.56 (0.35–0.87) **	1.31 (0.87–1.97)	0.71 (0.44–1.15)
€1000–2000/per month	1.28 (0.80–2.07)	1.25 (0.83–1.90)	0.77 (0.49–1.21)	1.63 (1.08–2.47) *	0.75 (0.47–1.22)
<€1000/per month	1.48 (0.86–2.53)	1.56 (0.98–2.50)	0.86 (0.52–1.43)	1.65 (1.03–2.65) *	1.15 (0.66–2.01)

Data are presented as adjusted odds ratio (OR) (95% confidence interval around OR). * *p* <0.05; ** *p* <0.01; *** *p* <0.001. ^a^ Continuous variable. ^†^ German equivalents to education levels listed in table from highest to lowest: high school equivalent = “Allgemeine Hochschulreife”, advanced technical college equivalent = “Fachhochschulreife”, secondary school equivalent = “Realschulabschluss”, junior high school equivalent = “Hauptschulabschluss”. Age units are based on DEBRA study participation eligibility (14 and older): 14–23; 24–33; 34–43; 44–53; 54–63; 64–73; 74–83; 84–93; 94–103.

**Table 4:** Multivariate associations with support (agree/totally agree) for the policy statements in the sample of current smokers (*n* = 586).

	Banning Sale of Tobacco	Raising Legal Age of Sale from 18 to 21	Taxing Tobacco Industry Sales	Assessing Effectiveness and Safety of E-Cigarettes	Banning Smoking in Cars When Minors Are Present
Cigarettes smoked/day, number ^a^	0.99 (0.95–1.03)	1.00 (1.00–1.00)	1.00 (1.00–1.00)	1.00 (1.00–1.00)	1.00 (1.00–1.00)
Quit attempt last year (yes/no)					
Yes, attempt to quit (reference)	1	1	1	1	1
No, attempt to quit	0.20 (0.10–0.40) ***	0.94 (0.59–1.50)	0.71 (0.45–1.11)	1.07 (0.69–1.65)	0.96 (0.61–1.52)
Motivation to stop smoking (MRS) ^b^	1.06 (0.95–1.18)	1.11 (1.02–1.20) *	1.00 (0.92–1.08)	1.01 (0.93–1.10)	1.02 (0.94–1.12)
Age, 10-year units ^a^	0.89 (0.73–1.10)	0.85 (0.75–0.96) **	0.92 (0.82–1.04)	0.98 (0.87–1.09)	1.01 (0.90–1.14)
Sex					
Female (ref.)	1	1	1	1	1
Male	0.58 (0.28–1.17)	0.84 (0.56–1.28)	0.93 (0.62–1.39)	0.76 (0.52–1.11)	0.56 (0.37–0.84) **
Education ^†^					
High school equiv. (ref.)	1	1	1	1	1
Advanced technical college equiv.	0.60 (0.07–5.36)	1.95 (0.69–5.52)	0.61 (0.21–1.76)	1.62 (0.52–5.02)	1.33 (0.43–4.15)
Secondary school equiv.	0.50 (0.19–1.35)	1.11 (0.60–2.05)	0.63 (0.37–1.09)	0.67 (0.39–1.15)	1.14 (0.65–2.00)
Junior high school equiv.	1.05 (0.39–2.80)	1.79 (0.95–3.36)	0.50 (0.28–0.89) *	0.63 (0.35–1.11)	1.44 (0.79–2.62)
No qualification	2.97 (0.45–19.82)	4.82 (1.31–17.79) *	0.73 (0.19–2.79)	0.47 (0.13–1.71)	1.88 (0.45–7.86)
Household income					
€>5000/per month (ref.)	1	1	1	1	1
€4000–5000/per month	1.37 (0.19–10.12)	0.97 (0.23–4.01)	0.22 (0.07–0.88) *	0.48 (0.14–1.69)	0.84 (0.19–3.81)
€3000–4000/per month	0.78 (0.14–4.40)	1.23 (0.41–3.72)	0.71 (0.25–1.98)	0.77 (0.28–2.11)	0.70 (0.21–2.39)
€2000–3000/per month	0.46 (0.09–2.53)	0.84 (0.29–2.50)	0.31 (0.11–0.85) *	0.92 (0.35–2.45)	0.48 (0.15–1.55)
€1000–2000/per month	0.57 (0.11–3.10)	1.22 (0.42–3.56)	0.47 (0.17–1.26)	1.30 (0.49–3.47)	0.44 (0.14–1.43)
<€1000/per month	0.53 (0.09–3.14)	1.17 (0.39–3.57)	0.34 (0.12–0.97) *	0.80 (0.29–2.20)	0.59 (0.17–1.98)

Data are presented as adjusted OR (95% confidence interval around OR). * *p* <0.05; ** *p* <0.01; *** *p* <0.001. ^a^ continuous variable, ^b^ continuous variable (increasing from 1 “do not want to stop” to 7 “really want to stop, intend to in the next month”). ^†^ German equivalents to education levels listed in table from highest to lowest: high school equivalent = “Allgemeine Hochschulreife”, advanced technical college equivalent = “Fachhochschulreife”, secondary school equivalent = “Realschulabschluss”, junior high school equivalent = “Hauptschulabschluss”. Age units are based on DEBRA study participation eligibility (14 and older): 14–23; 24–33; 34–43; 44–53; 54–63; 64–73; 74–83; 84–93; 94–103.

**Table 5:** Multivariate associations with support (agree/totally agree) for the policy statements in the sample of never- and ex-smokers (*n* = 1476).

	Banning Sale of Tobacco	Raising Legal Age of Sale from 18 to 21	Taxing Tobacco Industry Sales	Assessing Effectiveness and Safety of E-Cigarettes	Banning Smoking in Cars When Minors Are Present
Age, 10-year units ^a^	0.98 (0.92–1.05)	0.97 (0.91–1.03)	1.04 (0.98–1.12)	0.93 (0.87–0.99) *	0.92 (0.85–0.98) *
Sex					
Female (ref.)	1	1	1	1	1
Male	0.83 (0.65–1.10)	0.82 (0.66–1.02)	0.79 (0.62–1.00)	0.95 (0.75–1.20)	1.02 (0.79–1.31)
Education ^†^					
High school equiv. (ref.)	1	1	1	1	1
Advanced technical college equiv.	1.55 (0.95–2.53)	1.14 (0.72–1.81)	0.93 (0.56–1.53)	1.13 (0.70–1.84)	1.30 (0.74–2.29)
Secondary school equiv.	1.20 (0.86–1.66)	1.20 (0.88–1.60)	0.82 (0.59–1.13)	0.84 (0.61–1.14)	0.90 (0.64–1.27)
Junior high school equiv.	1.25 (0.87–1.79)	1.21 (0.87–1.68)	0.66 (0.46–0.94) *	0.87 (0.61–1.23)	0.97 (0.66–1.42)
No qualification	0.61 (0.19–1.98)	1.18 (0.43–3.25)	0.84 (0.26–2.74)	0.20 (0.10–0.68) *	0.64 (0.21–1.96)
Household income					
€>5000/per month (ref.)	1	1	1	1	1
€4000–5000/per month	1.00 (0.53–1.88)	1.16 (0.65–2.07)	0.88 (0.46–1.69)	1.44 (0.80–2.61)	0.81 (0.41–1.60)
€3000–4000/per month	0.72 (0.42–1.23)	1.00 (0.61–1.57)	0.66 (0.39–1.12)	1.24 (0.77–2.01)	0.75 (0.43–1.31)
€2000–3000/per month	1.17 (0.71–1.93)	1.15 (0.72–1.82)	0.62 (0.37–1.04)	1.35 (0.85–2.16)	0.82 (0.48–1.41)
€1000–2000/per month	1.34 (0.82–2.21)	1.23 (0.77–1.94)	0.84 (0.50–1.41)	1.60 (1.00–2.56)	0.96 (0.56–1.67)
<€1000/per month	1.64 (0.91–2.94)	1.84 (1.10–3.18)	1.23 (0.65–2.33)	2.38 (1.31–4.32) **	1.76 (0.86–3.60)

Data are presented as adjusted OR (95% confidence interval around OR). * *p* <0.05; ** *p* <0.01; ^a^ continuous variable, ^†^ German equivalents to education levels listed in table from highest to lowest: high school equivalent = “Allgemeine Hochschulreife”, advanced technical college equivalent = “Fachhochschulreife”, secondary school equivalent = “Realschulabschluss”, junior high school equivalent = “Hauptschulabschluss”. Age units are based on DEBRA study participation eligibility (14 and older): 14–23; 24–33; 34–43; 44–53; 54–63; 64–73; 74–83; 84–93; 94–103.
